# Supplementary material for: Novel Driver Strength Index highlights important cancer genes in TCGA PanCanAtlas patients
Source: PeerJ. 2022 Aug 11;10:e13860. doi: 10.7717/peerj.13860 (PMC9375969; doi:10.7717/peerj.13860)
Supplement: Supplemental Information 2 [file peerj-10-13860-s002.zip › COHORTS/genes plots/detailed/2021_8_16_14_9_distribution_events_detailed_30.pdf]

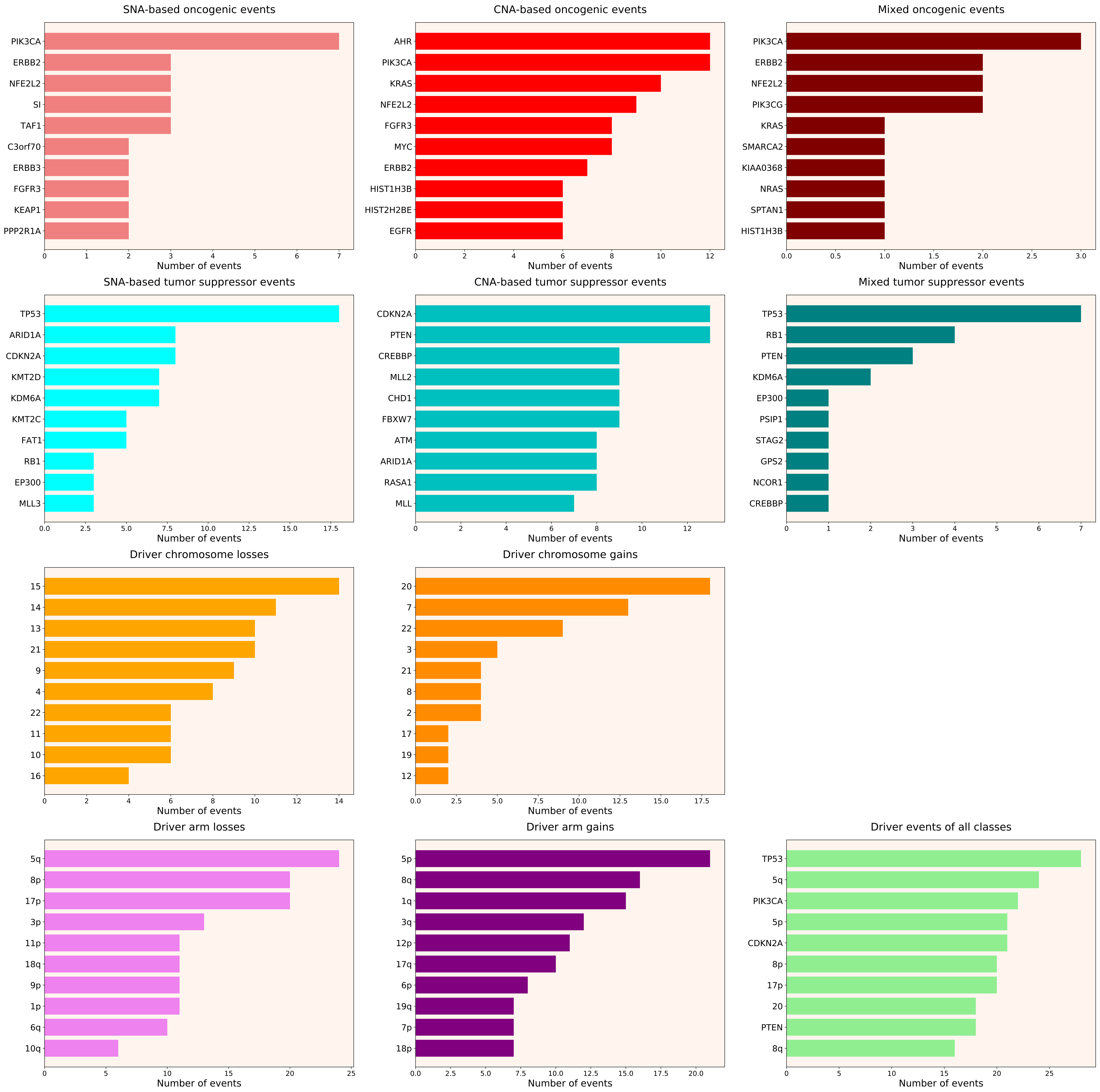

SNA-based oncogenic events
PIK3CA
ERBB2
NFE2L2
SI
TAF1
C3orf70
ERBB3
FGFR3
KEAP1
PPP2R1A
Number of events
CNA-based oncogenic events
AHR
PIK3CA
KRAS
NFE2L2
FGFR3
MYC
ERBB2
HIST1H3B
HIST2H2BE
EGFR
Number of events
Mixed oncogenic events
PIK3CA
ERBB2
NFE2L2
PIK3CG
KRAS
SMARCA2
KIAA0368
NRAS
SPTAN1
HIST1H3B
Number of events
SNA-based tumor suppressor events
TP53
ARID1A
CDKN2A
KMT2D
KDM6A
KMT2C
FAT1
RB1
EP300
MLL3
Number of events
CNA-based tumor suppressor events
CDKN2A
PTEN
CREBBP
MLL2
CHD1
FBXW7
ATM
ARID1A
RASA1
MLL
Number of events
Mixed tumor suppressor events
TP53
RB1
PTEN
KDM6A
EP300
PSIP1
STAG2
GPS2
NCOR1
CREBBP
Number of events
Driver chromosome losses
15
14
13
21
9
4
22
11
10
16
Number of events
Driver chromosome gains
20
7
22
3
21
8
2
17
19
12
Number of events
Driver arm losses
5q
8p
17p
3p
11p
18q
9p
1p
6q
10q
Number of events
Driver arm gains
5p
8q
1q
3q
12p
17q
6p
19q
7p
18p
Number of events
Driver events of all classes
TP53
5q
PIK3CA
5p
CDKN2A
8p
17p
20
PTEN
8q
Number of events
